# Rapid screening of *Salmonella enterica *serovars Enteritidis, Hadar, Heidelberg and Typhimurium using a serologically-correlative allelotyping PCR targeting the O and H antigen alleles

**DOI:** 10.1186/1471-2180-8-178

**Published:** 2008-10-09

**Authors:** Yang Hong, Tongrui Liu, Margie D Lee, Charles L Hofacre, Marie Maier, David G White, Sherry Ayers, Lihua Wang, Roy Berghaus, John J Maurer

**Affiliations:** 1 Department of Population Health, The University of Georgia, Athens, GA 30602, USA; 2 Department of Infectious Diseases, The University of Georgia, Athens, GA, USA; 3 Department of Statistics, The University of Georgia, Athens, GA 30602, USA; 4 Center for Food Safety and Quality Enhancement, The University of Georgia, Griffin, GA 30223, USA; 5 Center for Veterinary Medicine, U.S. Food and Drug Administration, Laurel, MD 20708, USA; 6 USDA ARS, Russell Research Center, 950 College Station road, Athens, GA 30605. T. Liu- Emory University, 1701 Uppergate Drive, Atlanta, GA 30322, USA

## Abstract

**Background:**

Classical *Salmonella *serotyping is an expensive and time consuming process that requires implementing a battery of O and H antisera to detect 2,541 different *Salmonella enterica *serovars. For these reasons, we developed a rapid multiplex polymerase chain reaction (PCR)-based typing scheme to screen for the prevalent *S. enterica *serovars Enteritidis, Hadar, Heidelberg, and Typhimurium.

**Results:**

By analyzing the nucleotide sequences of the genes for O-antigen biosynthesis including *wb*a operon and the central variable regions of the H1 and H2 flagellin genes in *Salmonella*, designated PCR primers for four multiplex PCR reactions were used to detect and differentiate *Salmonella *serogroups A/D1, B, C1, C2, or E1; H1 antigen types i, g, m, r or z_10_; and H2 antigen complexes, I: 1,2; 1,5; 1,6; 1,7 or II: e,n,x; e,n,z_15_. Through the detection of these antigen gene allele combinations, we were able to distinguish among *S. enterica *serovars Enteritidis, Hadar, Heidelberg, and Typhimurium. The assays were useful in identifying *Salmonella *with O and H antigen gene alleles representing 43 distinct serovars. While the H2 multiplex could discriminate between unrelated H2 antigens, the PCR could not discern differences within the antigen complexes, 1,2; 1,5; 1,6; 1,7 or e,n,x; e,n,z_15_, requiring a final confirmatory PCR test in the final serovar reporting of *S. enterica*.

**Conclusion:**

Multiplex PCR assays for detecting specific O and H antigen gene alleles can be a rapid and cost-effective alternative approach to classical serotyping for presumptive identification of *S. enterica *serovars Enteritidis, Hadar, Heidelberg, and Typhimurium.

## Background

There are approximately 15 cases of salmonellosis per 100,000 persons annually in the United States, more than double the 2010 Healthy People goal of 6.8 cases/100,000 individuals per year [1]. In order to reduce human illnesses, epidemiological measures have been implemented to reduce the source(s) of infection. Because food animals and poultry are recognized as important reservoirs of *Salmonella *[2,3], the United States Department of Agriculture (USDA) Food Safety Inspection Service (FSIS) implemented an "in plant" Hazard Analysis and Critical Control Point (HACCP) program to reduce the prevalence of foodborne pathogen contamination in meats, eggs, and milk. Although in-plant HACCP programs have been successful, further reductions in *Salmonella *contamination may require application of a risk reduction strategy to the farm environment. On-farm control programs have been effective in the past when they have been directed against vertically-transmitted *S. enterica *serovars (such as *S*. *enterica *serovar Enteritidis and *S*. *enterica *serovar Gallinarum) [4], but it is unclear whether this approach could be effective against all serovars. A more achievable goal may be to mitigate those *S. enterica *serovars that are most frequently associated with severe human illness. To further reduce *Salmonella *contamination in or on the final food product, producers may need to reduce its prevalence in animals brought into the meat processing plant. Producers may also need to accurately identify the source of *Salmonella *within a specific setting, in order to identify the points where an intervention [5] may be effective. Such an approach would require knowing whether these serovars are present on the farm. Also, determining the appropriate *S. enterica *serovar is a necessary first step in any epidemiological investigation of foodborne outbreaks; followed then by strain typing, using molecular based methods including pulsed-field gel electrophoresis (PFGE) [6] or amplified fragment length polymorphism that is needed to match patient strain to source [7]. Serotyping can be a formidable task because of the numerous antisera required and the expertise necessary for interpreting the agglutination reactions, thereby limiting its efficacy as a large scale screening tool.

There are currently 2,541 *S. enterica *serovars recognized based on antigenic differences in the lipopolysaccharide (LPS) O-antigen; and phase 1 (H1) and phase 2 (H2) flagellar antigens [8]. *Salmonella *can be further separated into monophasic and biphasic based on whether they express only one (H1) or both flagellar antigens (H1 and H2). The antigenic formula 4,5,12 (O): i (H1): 1,2 (H2) is the biphasic *S. enterica *serovar Typhimurium and 1,9,12 (O):g,m (H1):- (no H2) identifies the monophasic *S. enterica *serovar Enteritidis. Among the 2,541 *S. enterica *serovars identified to date, 10 *S. enterica *serovars: Typhimurium, Enteritidis, Newport, Heidelberg, Javiana, 4, [5], 12:i:-, Montevideo, Muenchen, Saintpaul, and Braenderup, currently account for 66% of all cases of laboratory-confirmed salmonellosis in the U.S. [8]. Between 1998–2006, *S. enterica *serovars Enteritidis, Hadar, Heidelberg, and Typhimurium also accounted for 48% of all *S. enterica *serovars isolated from poultry, including chicken broilers, ground chicken and ground turkey, in the U.S. [9]. Worldwide, two serovars, Enteritidis and Typhimurium are responsible for 79% of reported cases of salmonellosis [10].

*Salmonella *serotyping is based on the identification of the variable O and H antigens. Because the antigenic composition of the O, H1 and H2 antigens are a reflection of their unique DNA sequence alleles [11,12], PCR and similar nucleotide-based methods have made it possible to accelerate the identification of serotypes based upon the identification of unique genes or gene arrangements [13-18] and use as a diagnostic tool [19]. We report here on the development and validation of a serologically-correlative PCR-based assay that could solve a number of the logistical challenges faced by diagnostic and food microbiology labs.

## Results and discussion

### Multiplex PCR differentiation of *Salmonella enterica *serovars Enteritidis, Hadar, Heidelberg, and Typhimurium

**Figure 1:**
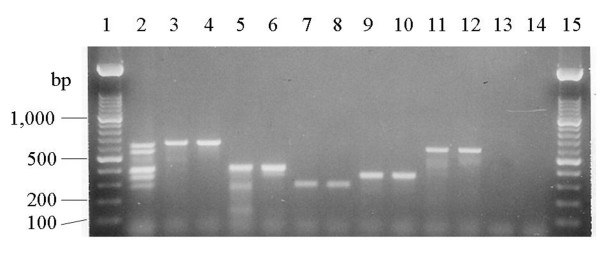
**Multiplex PCR for identifying serogroup-specific, *Salmonella *O antigen biosynthesis gene(s)**. Lanes1 and 15: 100 bp MW standard; lane 2, multiplex PCR control for five *Salmonella *serogroups; lane 3: *S. enterica *serovar Paratyphi A [A]; lane 4: *S. enterica *serovar Enteritidis [D1]; lane 5: *S. enterica *serovar Muenchen [C2]; lane 6: *S. enterica *serovar Hadar [C2]; lane 7: *S. enterica *serovar Anatum [E1]; lane 8: *S. enterica *serovar London [E1]; lane 9: *S. enterica *serovar Infantis [C1]; lane 10: *S. enterica *serovar Tennessee [C1]; lane 11: *S. enterica *serovar Saintpaul [B]; lane 12, *S. enterica *serovar Typhimurium [B]; lane 13: *E. coli *K12 LE392, negative control; and lane 14: no DNA control. The sizes of the PCR amplicons are 624 bp for serogroup A/D1, 561 bp for serogroup B, 341 bp for serogroup C1, 397 bp for serogroup C2, and 281 bp for serogroup E1.

**Figure 2:**
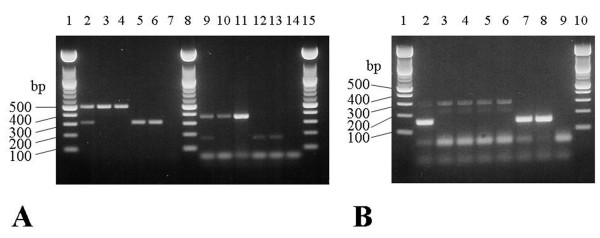
**Multiplex PCR for identifying *Salmonella *H1 and H2 gene alleles**. ***(a) ***Multiplex PCR for identifying H1 antigen gene alleles: i, g,m, r, and z_10_. Lanes 2–7: H1-1 multiplex PCR for i and g,m antigens. Lanes 9–14: H1-2, multiplex PCR for antigens r and z10. Lanes 1, 8, and 15: 100 bp MW standard; lane 2: H1-1 multiplex PCR control; lane 3: *S. enterica *serovar Typhimurium [i]; lane 4: *S. enterica *serovar Kentucky [i]; lanes 5 and 6: *S. enterica *serovar Enteritidis [g,m]; lane 7: no DNA control; lane 9: H1-2 multiplex PCR control; lane 10: *S. enterica *serovar Hadar [z_10_]; lane 11: *S. enterica *serovar Mbandaka [z_10_]; lane12: *S. enterica *Heidelberg [r]; lane 13: *S. enterica *serovar Infantis [r]; and lane 14: no DNA control. The sizes of the PCR amplicons are: 508 bp for i, 309 bp for g,m, 169 bp for r, and 363 bp for z_10_. ***(b) ***Multiplex PCR for identifying H2 antigen complexes I: 1,2, 1,5, 1,6, 1,7 and II: e,n,x, e,n,z_15 _respectively. Lanes 1 and 10: 100 bp MW standard; lane 2: multiplex PCR control for H2 antigen complexes I: 1,2; 1,5; 1,6; 1,7 and II: e,n,x; e,n,z_15_; lane 3: *S. enterica *serovar Typhimurium [1,2]; lane 4: *S. enterica *serovar Infantis [1,5]; lane 5: *S. enterica *serovar Anatum [1,6]; lane 6, *S. enterica *serovar Bredeney [1,7]; lane 7: *S. enterica *serovar Hadar [e,n,x]; lane 8: *S. enterica *serovar Mbandaka [e,n,z_15_]; and lane 9: no DNA control. The sizes of the PCR amplicons are 294 bp for H2 antigen complex I: 1,2; 1,5; 1,6; 1,7 and 152 bp for H2 antigen complex II: e,n,x and e,n,z_15_.

We developed multiplex PCRs targeted to the O, H1, and H2 alleles associated with four *S. enterica *serovars Enteritidis, Hadar, Heidelberg, and Typhimurium. Specific PCR primers to identify specific *Salmonella *serogroups, H1 and H2 alleles were designed based on the divergence of the glycosyl synthase genes, the unique linkage between two genes for a specific O-antigen of *Salmonella*, or allele-specific sequences within the hypervariable region of H1 and H2 antigen genes. In the primer design, a unique amplicon size was selected in order to facilitate development of a multiplex PCR (Table 1, Fig. 1 &2). The ability of the multiplex PCR to correctly identify serogroups (Fig. 1) was evaluated for 239 *Salmonella *isolates representing forty-three different serotypes which belonged to one of the six major serogroups, A, B, C1, C2, D1 and E1. With the exception of serogroups A and D1, which produce the same size amplicons (Kappa = 0.98), the multiplex PCR accurately distinguished salmonellae belonging to serogroups B, C1, C2, and E1 (Kappa = 1.00) (Table 2). The inability to distinguish serogroups A and D1 is due to the high degree of nucleotide sequence homology between the *prt *(paratose synthase) genes [20]. The *fliC *multiplex PCRs successfully detected the H1, i, r, or z_10_, alleles (Fig. 2A) and no amplicons were produced for serovars with other H1, flagellins (Kappa = 1.00) (Table 2). However, the *fliC *g,m primer set produced the same size amplicon only for salmonellae that possessed both the g and m, or g alone, or either epitope, g or m, in combination with other serotype-specific epitopes, or non-motile salmonellae that possess the *fliC *g,m allele [21] and therefore it did not have the specificity of the other H1 primer sets (Kappa = 0.58 vs. 1.00) (Table 2). To complement our PCR-based H allelotyping, a *fljB *multiplex PCR was designed to detect the H2 antigen alleles by targeting conserved regions within *fljB *alleles encoding the antigen complexes I: 1,2; 1,5; 1,6; 1,7 or II: e,n,x; e,n,z_15 _and producing unique size amplicons (Table 1, Fig. 2B). The expected size amplicons were produced for only those *S. enterica *serovars belonging to H2 antigen complexes I: 1,2; 1,5; 1,6: 1,7 and. II: e,n,x; e,n,z_15 _(Fig. 2B). The H2 multiplex PCR however could not distinguish H2 1,2 allele (Kappa = 0.75) or e,n,x (Kappa = 0.54) among the different H2 alleles within each antigen complex; for example indistinguishable amplicons were produced for *Salmonella *isolates bearing 1,2 vs 1,5; 1,6; or 1,7 (Table 2).

**Table 1:** Primers used for multiplex PCR to detect and differentiate *Salmonella enterica *serogroups and serovars

**Target gene**^1^	**Nucleotide sequence**	**Expected Size (bp)**
O-antigen multiplex		
*abe*_1 _(B)	F: GGCTTCCGGCTTTATTGG	561
	R: TCTCTTATCTGTTCGCCTGTTG	
*wbaD-manC *(C1)	F: ATTTGCCCAGTTCGGTTTG	341
	R: CCATAACCGACTTCCATTTCC	
*abe*_2 _(C2)	F: CGTCCTATAACCGAGCCAAC	397
	R: CTGCTTTATCCCTCTCACCG	
*prt *(A/D1)	F: ATGGGAGCGTTTGGGTTC	624
	R: CGCCTCTCCACTACCAACTTC	
*wzx *– *wzy *(E1)	F: GATAGCAACGTTCGGAAATTC	281
	R: CCCAATAGCAATAAACCAAGC	

H1-1 multiplex		
*fliC *(i)	F: AACGAAATCAACAACAACCTGC	508
	R: TAGCCATCTTTACCAGTTCCC	
*fliC *(g,m)	F: GCAGCAGCACCGGATAAAG	309
	R: CATTAACATCCGTCGCGCTAG	

H1-2 multiplex		
*fliC *(r)	F: CCTGCTATTACTGGTGATC	169
	R: GTTGAAGGGAAGCCAGCAG	
*fliC *(z_10_)	F: GCACTGGCGTTACTCAATCTC	363
	R: GCATCAGCAATACCACTCGC	

H2 multiplex		
*fljB *(I: 1,2; 1,5; 1,6; 1,7)	F: AGAAAGCGTATGATGTGAAA	294
	R: ATTGTGGTTTTAGTTGCGCC	
*fljB *(II: e,n,x; e,n,z_15_)	F: TAACTGGCGATACATTGACTG	152
	R: TAGCACCGAATGATACAGCC	

^1^Indicates the unique genes or the junctions between the two genes used for designing PCR primers. () = antigen(s) detected.

**Table 2:** Comparison of multiplex PCR to serotyping for identifying *Salmonella *O alleles B; C1; C2; D1 or E1; H1 alleles i; g,m; r or z_10_;and H2 alleles 1,2 or e,n,x

										**Phase 1 PCRs**				**Phase 2 multiplex PCR**	
												
**Antigenic Formula**					**O muliplex PCR**					**i/g,m multiplex PCR**		**r/z**_10_**multiplex PCR**			
			
**O**	**H1**	**H2**	***S. enterica *Serovars**	**Animal Origin (*n*)**^1^	**B**	**C1**	**C2**	**D1**	**E1**	**i**	**g,m**	**r**	**z**_10_	**1,2**	**e,n,x**
A	a	1,5	Paratyphi A	1 (1)	0	0	0	1	0	0	0	0	0	1	0
B	b	1,2	Paratyphi B	1 (1)	1	0	0	0	0	0	0	0	0	1	0
B	e,h	1,2	Saintpaul	1(1)	1	0	0	0	0	0	0	0	0	1	0
B	e,h	1,5	Reading	1(2)	2	0	0	0	0	0	0	0	0	1	0
B	f,g	-	Derby	1(1)	1	0	0	0	0	0	1	0	0	0	0
B	i	1,2	Typhimurium	1, 4–6 (74)	74	0	0	0	0	74	0	0	0	74	0
B	l,v	1,7	Bredeney	1(1)	1	0	0	0	0	0	0	0	0	1	0
B	1,v	e,n,z_15_	Brandenburg	1(2)	2	0	0	0	0	0	0	0	0	0	2
B	b	-	Java	6(1)	1	0	0	0	0	0	0	0	0	0	0
B	e,h	e,n,x	Chester	1(2)	2	0	0	0	0	0	0	0	0	0	2
B	f,g,s	-	Agona	1 (1)	1	0	0	0	0	0	1	0	0	0	0
B	r	1,2	Heidelberg	1, 3–6(24)	24	0	0	0	0	0	0	24	0	24	0
B	z	1,5	Kiambu	1(1)	1	0	0	0	0	0	0	0	0	1	0
B	z	1,7	Indiana	1(2)	2	0	0	0	0	0	0	0	0	2	0
B	z_10_	1,2	Haifa	6 (1)	1	0	0	0	0	0	0	0	1	1	0
C1	b	l,w	Ohio	1(1)	0	1	0	0	0	0	0	0	0	0	0
C1	c	1,5	Choleraesuis	1, 6(6)	0	6	0	0	0	0	0	0	0	6	0
C1	c	1,5	Paratyphi C	1 (1)	0	1	0	0	0	0	0	0	0	1	0
C1	d	l,w	Livingstone	6(1)	0	1	0	0	0	0	0	0	0	0	0
C1	g,m,s	-	Montevideo	1, 5(12)	0	12	0	0	0	0	12	0	0	0	0
C1	k	1,5	Thompson	1(1)	0	1	0	0	0	0	0	0	0	1	0
C1	m,t	-	Oranienburg	1(1)	0	1	0	0	0	0	1	0	0	0	0
C1	z_29_	-	Tennessee	1(1)	0	1	0	0	0	0	0	0	0	0	0
C1	e,h	e,n,z_15_	Braenderup	1(2)	0	2	0	0	0	0	0	0	0	0	2
C1	r	1,5	Infantis	1(2)	0	2	0	0	0	0	0	2	0	2	0
C1	z_10_	e,n,z_15_	Mbandaka	1(14)	0	14	0	0	0	0	0	0	14	0	14
C1	z_28_	-	Lille	1(1)	0	1	0	0	0	0	0	0	0	0	0
C2	d	1,2	Muenchen	5(3)	0	0	3	0	0	0	0	0	0	3	0
C2	e,h	1,2	Newport	4,5(1)	0	0	1	0	0	0	0	0	0	1	0
C2	i	z_6_	Kentucky	1(24)	0	0	24	0	0	24	0	0	0	0	0
C2	z_10_	e,n,x	Hadar	1 (10)	0	0	10	0	0	0	0	0	10	0	10
D1	a	1,5	Miami	5(1)	0	0	0	1	0	0	0	0	0	1	0
D1	a	1,5	Sendai	5(1)	0	0	0	1	0	0	0	0	0	1	0
D1	g,m	-	Enteritidis	1(20)	0	0	0	20	0	0	20	0	0	0	0
D1	g,p	-	Dublin	2, 6(3)	0	0	0	3	0	0	3	0	0	0	0
D1	l,v	1,5	Panama	1 (1)	0	0	0	1	0	0	0	0	0	1	0
D1	-	-	Gallinarum	1(4)	0	0	0	4	0	0	4	0	0	0	0
D1	f,g,t	-	Berta	1 (2)	0	0	0	2	0	0	2	0	0	0	0
D1	l,z_28_	1,5	Javiana	1 (1)	0	0	0	1	0	0	0	0	0	1	0
E1	e,h	1,5	Muenster	1(2)	0	0	0	0	2	0	0	0	0	2	0
E1	l,v	1,7	Give	1(2)	0	0	0	0	2	0	0	0	0	2	0
E1	e,h	1,6	Anatum	1, 5 (4)	0	0	0	0	4	0	0	0	0	4	0
E1	l,v	1,6	London	1(2)	0	0	0	0	2	0	0	0	0	2	0
		Total		239	114	43	38	34	10	98	44	26	25	135	30
		False Positives			0	0	0	1	0	0	24	0	0	30	18
		False Negatives			0	0	0	0	0	0	0	0	0	0	0
		Kappa^2^			1.0	1.0	1.0	0.98	1.0	1.0	0.58	1.0	1.0	0.75	0.54

^1^1 = poultry; 2 = bovine; 3 = swine, 4 = other (includes dog, heron, horse, opossum, parrot, rabbit, and snake); 5 = human; and 6 = unknown. Numbers in parentheses indicate the numbers of isolates for each serovar.

^2^Agreement between PCR allelotyping and conventional serotyping results

### Comparison of multiplex PCR allelotyping of O, H1, and H2 genes with conventional serotyping in differentiating *S. enterica *serovars Enteritidis, Hadar, Heidelberg and Typhimurium

Validation of the allelotyping method is important for its integration with conventional *Salmonella *culture and typing methods used in diagnostic and food microbiology [22-25]. We therefore assessed the allelotyping multiplex PCR against the standard conventional *Salmonella *serotyping method in identifying *Salmonella *O, H1 and H2 antigens for 43 different serovars of salmonellae isolated mainly from chicken carcasses and poultry environments (Tables 2 and 3).

**Table 3:** Allelotyping PCR scheme for presumptive identification of *S. enterica *serovars Enteritidis, Hadar, Heidelberg, and Typhimurium

**O-multiplex**	**H1-multiplexes**^1^	**H2-multiplex**	**Serovars**	**Sensitivity**^5^	**Specificity**^5^
B	i	I^2^	Typhimurium	1.00	1.00
B	r	I	Heidelberg	1.00	1.00
C2	z_10_	II^3^	Hadar	1.00	1.00
A/D1	g,m	-^4^	Enteritidis	1.00	0.96

^1^Identifies H1 alleles i; g,m; r; or z_10_

^2 ^Covers H2 alleles 1,2; 1,5; 1,6; and 1,7

^3 ^Covers H2 alleles e,n,x and e,n,z_15_

^4 ^PCR negative for H2-multiplex

^5^Sensitivity and specificity of the allelotyping PCR scheme relative to conventional serotyping in identifying *S. enterica *serovars Enteritidis, Hadar, Heidelberg, and Typhimurium among the 239 isolates examined in this study.

The allelotyping PCR scheme for identifying *S. enterica *serovars Enteritidis, Hadar, Heidelberg and Typhimurium is envisioned to work as follows. An initial multiplex PCR is performed to determine which O antigen allele that an isolate possesses and a serogroup designation is given or unknown, based on PCR results. If the isolate possesses O alleles for serogroups B, C2, or A/D1, then a 2^nd ^allelotyping PCR is done to determine the presence of H2 alleles: i; g,m; r; or z_10_. Based on the results of this 2^nd ^allelotyping PCR, an H1 allele type can be given an isolate as either being i; g,m; r; z_10 _or unknown, if no amplicons with the expected size for the H1 allelotyping PCR are produced. If both O and H1 allelotyping PCR detects O and H1 alleles associated with *S. enterica *serovars Enteritidis, Hadar, Heidelberg, and Typhimurium, then a 3^rd ^final H2 allelotyping PCR is performed to further differentiate the isolate to serovar level. Therefore, identifying one allele for each O, H1, and H2 allelotyping PCR, as listed in Table 3; it is possible to discern the serovar for isolates typed using this PCR-based scheme. For example, identification of serogroup B, H1 i, and H2 I antigen complex by multiplex PCR presumptively identifies the isolate as *S. enterica *serovar Typhimurium (Sensitivity = 1.00; Specificity = 1.00) (Table 3). The expansion of O-antigen PCR to detect serogroups C1 and E1, affords a laboratory the opportunity to detect other *S. enterica *serovars, as the antigenic formula for O, H1 and H2 antigens defines the serovar. Therefore, we were able to identify additional *S. enterica *serovars with our multiplex PCRs including Haifa [B; z_10_; 1,2], Infantis [C1; r; 1,5], and Mbandaka [C1; z_10_, e,n,z_15_]. We can also identify monophasic *S. enterica *serovars (ex. Montevideo: [C1; g,m,s; -]) by including a generic *Salmonella fljB *(H2) PCR test [14]. Isolates negative for O, H1, and H2 alleles by our multiplex PCR screen would need to be characterized by traditional serotyping, RFLP PCR [14], or PCR screens that identify the other H1 and H2 alleles [15,16,22]. The limitations with our multiplex PCR are that it cannot distinguish among serogroup/serovar variants that arise due to phage conversion and the resulting chemical/antigenic alteration of the somatic O antigen [8] (ex. Hadar vs. Istanbul), or subtle point mutations in H2 antigen gene, *fljB *responsible for loss of flagellar expression observed in some *S. enterica *serovar Typhimurium strains [26]. Our multiplex PCRs were designed to be used as a rapid screen for *S. enterica *serovars: Enteritidis, Hadar, Heidelberg, and Typhimurium, targeting key genes/alleles that differentiate these serovars from the rest. As a diagnostic test, our allelotyping PCR was also designed to minimize the cost of this test to a few individual PCR tests, with a minimum number of primers needed for this typing scheme. Unfortunately, our H2 multiplex PCR cannot discern differences within the H2 antigen complexes (Table 2) to make a definitive serovar designation for *S. enterica *serovars with the same O and H1 antigens as our target serovars (*S. enterica *serovars: Typhimurium [H2: 1,2] vs. Lagos [H2: 1,5]; Heidelberg [H2: 1,2] vs. Bradford [H2: 1,5], Winneba [H2: 1,6] or Remo [H2: 1,7]; or Hadar [H2: e,n,x] vs. Glostrup [H2: e,n,z15]). Also, the allelotyping primers for H1 g,m allele identifies those H1 alleles bearing g or m in any possible combination (Table 2), therefore H1 multiplex would not be able to discern serogroup D1, *S. enterica *serovars Enteritidis [H1: g,m] from Blegdam [g,m,q]. While the possibility of encountering these alternate serovars may be remote based on epidemiological data [8,9], it is still a possibility, and where a reporting laboratory may require confirmatory testing there are additional PCR based tests that can discern these allelic differences to make a final, definitive serovar designation possible [15,16]. Alternatively, the H2 amplicons can be sequenced to definitively identify the H2 allele. Although several multiplex PCRs have been developed to assist laboratories in identification of *S. enterica *serovars [15-17,22], our results are the first to focus on, validate and describe a PCR-based scheme for assisting diagnostic labs in differentiating *S. enterica *serovars: Enteritidis, Hadar, Heidelberg, and Typhimurium.

## Conclusion

The conventional *Salmonella *serological serotyping scheme is a time-consuming, labor-intensive and expensive procedure. With this PCR based allelotyping scheme, specific *S. enterica *serovars can be differentiated rapidly. The method is cost-effective and needs little technical training. This multiplex PCR allows large service laboratories to rapidly identify *S. enterica *serovars of public health importance including Enteritidis, Hadar, Heidelberg, and Typhimurium and focus conventional efforts towards identification of unusual serovars.

## Methods

### Bacterial strains

The *S. enterica *isolates used in this study were from multiple animal species, including human, poultry, livestock and wildlife [27-30], and serotyped by the National Veterinary Service Laboratory (NVSL; Ames, IA) using classical methods (Table 2). The isolates were used to test the specificity of PCRs specific for O, H1 and H2 alleles described in Table 1. Additional *Salmonella *isolates of unknown serovars were obtained from two poultry farms in northeast Georgia [25,31] as well as salmonellae isolated from routine submissions to the Poultry Diagnostic and Research Center (PDRC) in Athens, GA.

### Isolation and serotyping of *Salmonella*

We sampled the commercial chicken broiler house environment and chicken carcasses for *Salmonella *as previously described [31]. The processing, enrichment, isolation and final diagnostic confirmation of *Salmonella *from samples is described in detail elsewhere [31]. Serotyping was done using standard serological typing procedures for *Salmonella *O, H1 and H2 antigens [32].

### PCR primer design

From comparative analysis of the *wba *operon for *Salmonella *serogroups A/D1, B, C1, C2, and E1 [20,33-37] we identified serogroup-specific gene(s) (National Center for Biotechnology Information (NCBI) Accession #: M29682, X56793, X61917, M84642, X60665) for PCR primer design. Similarly, we identified from an alignment within the central variable region [11,38,39] of *fliC *(H1) and *fljB *(H2) alleles (NCBI accession #: D13689, M84974, AF15949, AF332601, U06199, U06206, U06225, U06197, M84973, Z15086, D78639, Z15071, Z15072, Z15069, U06205, U06204, AF420426, AF420425, AF045151, U17175, U17171, U17172, AF425736, AF425737), using the DNA analysis software AlignPlus^® ^version 3.0 (Scientific and Educational Software), candidate sequences to differentiate *Salmonella *with the H1 flagellin antigens i, g,m, r, z_10_, and the H2 flagellin antigen complexes 1,2, 1,5, 1,6, 1,7 and e,n,x, e,n,z_15 _alleles. We analyzed these gene sequences, using the GeneRunner^® ^(Hastings software; Hastings, NY) DNA analysis software, and identified suitable primer sets that were compatible in a single multiplex PCR reaction and designed to produce an amplicon with size unique for the sequence(s) targeted by a specific primer set (Table 1).

### Multiplex allelotyping PCR for Salmonella O, H1, and H2 antigen genes and differentiating *S. enterica *serovars Enteritidis, Hadar, Heidelberg, and Typhimurium

The O-antigen multiplex PCR was designed to detect serogroup A/D1, B, C1, C2, or E1 specific genes or alleles (Table 1). The O-antigen multiplex PCR was performed using the Amplitron II Thermolyne thermocycler (Barnstead; Dubuque, IA), using HotStart PCR tubes (Molecular Bio-Products, Inc., San Diego, CA). Each reaction had a final concentration of 1.5 mM MgCl_2_, 50 mM Tris, pH 8.3, 0.25 mg/ml bovine serum albumin, 0.5 μM primer, 0.2 mM deoxynucleotides (Boehringer Mannheim; Indianapolis, IN), 0.5 units of *Taq *DNA polymerase (Boehringer Mannheim), and 1 μl of whole cell template. The PCR was performed with pre-amplification heating as described by D'Aquilla et al. [40]. The program parameters for PCR include an initial five minutes incubation at 85°C, to mix the two PCR reaction mixes, followed by 30 cycles of denaturation (94°C for 1 min), annealing (55°C for 1 min), and extension (72°C for 1 min). Amplicons were separated on 1.5% agarose gel with Tris-acetate-EDTA buffer [41] and ethidium bromide (0.2 μg/ml) at 100 V. The 100-bp ladder (GIBCO/BRL, Gaithersburg, MD) was used as a molecular weight (MW) standard for determining the MW of the PCR products. Various *S. enterica *serovars belonging to serogroups A/D1, B, C1, C2, E1 were used in the PCR to test the specificity of the primer sets.

The H1-1 multiplex PCR was used to identify isolates with antigens i or g, m; while the H1-2 multiplex PCR was designed to detect isolates with antigens r or z_10_. Finally, the H2 multiplex PCR was created to differentiate isolates with either H2 antigen complexes 1,2; 1,5; 1,6; 1,7; or e,n,x; e,n,z_15_. In order to identify the H1 and H2 alleles, capillary PCR reaction was performed to amplify the alleles of *fliC *and *fljB *by three multiplex PCRs with the Rapidycler™ hot-air thermocycler (Idaho Technologies; Idaho Falls, ID) [42] in 10-μl capacity capillary tubes. We sought to reduce the expense of reagents and reaction time by utilizing a capillary thermocycler that accommodates very low reaction volumes. The 10-μl PCR mix for the *fliC *i and g,m multiplex consisted of 2.0 mM MgCl_2_, 50 mM Tris (pH 8.3), 0.25 mg/ml bovine serum albumin, 0.5 μM of each primer, 0.2 mM deoxynucleotides, 5% DMSO, 1.0 units of *Taq *DNA polymerase, and 1 μl whole cell template. For *fliC *r and z_10 _multiplex, 3.0 mM MgCl_2 _and 1.0 μM of each primer were used for each reaction. For the amplification of the H2 alleles, the *fljB *multiplex consisted of 3.75 mM MgCl_2_, 62.5 mM Tris, pH 8.3, 0.31 mg/ml bovine serum albumin, 0.5 μM of each primer, 0.2 mM deoxynucleotides, 5% DMSO, 1.0 units of *Taq *DNA polymerase, and 1 μl whole cell template in a 10 μl volume. The program parameters for the hot-air thermocycler were an initial heating step of 94°C for 1 min; 94°C for 1 sec, 55°C for 1 sec, and 72°C for 20 sec with a slope of 2.0 for 40 cycles; and a final extension at 72°C for 4 min. Amplicons were detected as described above. The specificity of the PCR detection was tested against various *Salmonella *serovars possessing the relevant *fliC *and *fljB *alleles (Table 2). *Escherichia coli *LE392 served as a negative control. Whole cell template for all multiplex PCRs was prepared according to the procedures of Hilton et al. [43].

### Statistics

Kappa statistics were calculated to evaluate the agreement between the classical serotyping systems and multiplex PCR for each of the antigen groups examined. Sensitivity and specificity of the allelotyping PCR scheme relative to conventional serotyping was calculated for *S. enterica *serovars Enteritidis, Hadar, Heidelberg, and Typhimurium.

## Abbreviations

CDC: Center for Disease Control and Prevention; FSIS: Food Safety Inspection Service; HACCP: Hazard Analysis and Critical Control Point; NCBI: National Center for Biotechnology Information; NPIP: National Poultry Improvement Plant; PCR: polymerase chain reaction; PFGE: pulsed-field gel electrophoresis; RFLP: restriction fragment polymorphism; SNP: single nucleotide polymorphism; USDA: United States Department of Agriculture.

## Authors' contributions

JJM designed, directed, and supervised most aspects of this project. YH designed, and optimized the multiplex PCRs described in this study, as well as wrote the first draft of this manuscript. MDL and CH were involved in translation of these molecular tests to the diagnostic lab. CH was instrumental in our access to poultry farms and companies to obtain samples/isolates for testing. TL assessed the multiplex PCR in identifying *S. enterica *serovars for isolates submitted to the PDRC diagnostic lab. MDL and DW were involved in instruction, supervision, and interpretation of classical serotyping. MM and SA assisted in conventional serotyping of isolates. LW did statistical analyses of PCR vs. classical serotyping. RB evaluated and interpreted the statistical tests. MM, TL, and SA roles in this study were beyond those normally associated with their jobs and the University of Georgia or FDA. All authors have read and approved the final manuscript.
